# Phosphorylation of the compartmentalized PKA substrate TAF15 regulates RNA–protein interactions

**DOI:** 10.1007/s00018-024-05204-4

**Published:** 2024-04-03

**Authors:** Andreas Feichtner, Florian Enzler, Valentina Kugler, Katharina Hoppe, Sophia Mair, Leopold Kremser, Herbert Lindner, Roland G. Huber, Ulrich Stelzl, Eduard Stefan, Omar Torres-Quesada

**Affiliations:** 1 Tyrolean Cancer Research Institute (TKFI), Innrain 66, 6020 Innsbruck, Austria; 2 Institute of Molecular Biology and Center for Molecular Biosciences, University of Innsbruck, Technikerstrasse 25, 6020 Innsbruck, Austria; 3 Daniel Swarovski Research Laboratory, Department of Visceral, Transplant and Thoracic Surgery, Medical University of Innsbruck, Innrain 66/66a, 6020 Innsbruck, Austria; 4 Institute of Developmental Immunology, Biocenter, Medical University of Innsbruck, Innrain 80/82, 6020 Innsbruck, Austria; 5 Department of Cardiac Surgery, Medical University of Innsbruck, Innrain 66/66a, 6020 Innsbruck, Austria; 6 Vascage, Center of Clinical Stroke Research, 6020 Innsbruck, Austria; 7 Division of Clinical Biochemistry, Biocenter, Medical University of Innsbruck, Innrain 80/82, 6020 Innsbruck, Austria; 8 Bioinformatics Institute, Agency for Science Technology and Research, Singapore, 138671 Singapore; 9 Institute of Pharmaceutical Sciences, University of Graz, Schubertstrasse 1, 8010 Graz, Austria; 10 Division of Medical Biochemistry, Biocenter, Medical University of Innsbruck, Innrain 80/82, 6020 Innsbruck, Austria

**Keywords:** Molecular interactions, Neurodegeneration, FET-protein, ALS, Colon cancer, Interaction network, Proteomics, Affinity purification, Post-translational modification

## Abstract

Spatiotemporal-controlled second messengers alter molecular interactions of central signaling nodes for ensuring physiological signal transmission. One prototypical second messenger molecule which modulates kinase signal transmission is the cyclic-adenosine monophosphate (cAMP). The main proteinogenic cellular effectors of cAMP are compartmentalized protein kinase A (PKA) complexes. Their cell-type specific compositions precisely coordinate substrate phosphorylation and proper signal propagation which is indispensable for numerous cell-type specific functions. Here we present evidence that TAF15, which is implicated in the etiology of amyotrophic lateral sclerosis, represents a novel nuclear PKA substrate. In cross-linking and immunoprecipitation experiments (iCLIP) we showed that TAF15 phosphorylation alters the binding to target transcripts related to mRNA maturation, splicing and protein-binding related functions. TAF15 appears to be one of multiple PKA substrates that undergo RNA-binding dynamics upon phosphorylation. We observed that the activation of the cAMP-PKA signaling axis caused a change in the composition of a collection of RNA species that interact with TAF15. This observation appears to be a broader principle in the regulation of molecular interactions, as we identified a significant enrichment of RNA-binding proteins within endogenous PKA complexes. We assume that phosphorylation of RNA-binding domains adds another layer of regulation to binary protein-RNAs interactions with consequences to RNA features including binding specificities, localization, abundance and composition.

**Supplementary Information:**

The online version contains supplementary material available at 10.1007/s00018-024-05204-4.

## Introduction

Cell surface receptors sense, transform and relay the pandemonium of extracellular signals through intracellular enzyme cascades. In many cases this involves the activation and inactivation of signaling complexes which are composed of compartmentalized protein kinase units [1–3]. The dynamic modulation of these signaling cascades requires precise molecular interactions (i.e. protein–protein, protein-RNA/DNA and protein-small molecule-interactions) which are tightly regulated and localized to explicit subcellular compartments to control the cellular responses [4, 5]. Input signals received at the cell surface are converted to intracellular second messenger fluxes [6, 7]. These second messenger pulses modulate the activities of the signaling enzymes spatiotemporally by relaying post-translational modifications (PTMs) and altering the formation of protein-complexes [8, 9].

The 3′, 5′-cyclic adenosine monophosphate (cAMP) is a ubiquitous cellular second messenger molecule at multiple convergence points of intracellular signaling pathways [10, 11]. cAMP pools are generated upon activation of G protein-coupled receptors (GPCRs) which are coupled to the adenylyl cyclase (AC) stimulatory (Gs) or inhibitory (Gi) G-proteins, respectively [8, 12–15]. ACs catalyze the conversion of ATP to cAMP. The increase of intracellular cAMP levels leads to the activation of its main effector protein complex, the protein kinase A (PKA) [16]. Inactive PKA forms a tetrameric complex, composed of two regulatory and two catalytic subunits. Binding of cAMP to the regulatory subunits leads to release and activation of the catalytic subunits and subsequent phosphorylation of substrate proteins [17, 18]. Phosphodiesterase enzymes hydrolize cAMP and thereby limit its zone of influence to distinct and highly localized microdomains, which constitute the frame wherein PKA substrate phosphorylation occurs [19]. A-kinase anchoring proteins (AKAPs) form the backbone of PKA mediated signal propagation by binding to the regulatory subunits and tethering the PKA holoenzyme in the vicinity of its substrates [20–23]. PKA targets a broad variety of substrates, and represents one of the best studied examples for dynamic protein–protein and protein-small molecule-interactions. The cAMP-PKA pathway plays a key role in different physiological processes such as cell-proliferation, differentiation, and metabolism [24]. On the cellular level, PKA is centrally involved in glucose and lipid metabolism, and mitochondrial functions [25, 26]. Deregulation of different layers of the cAMP-PKA signaling axis is associated with a variety of diseases as it has been recently summarized [15]. Hence, a deeper understanding of the physiological and pathological compartmentalized PKA signal propagation is needed.

Post-transcriptional gene regulation is essential to maintain cellular metabolism, coordinate maturation, transport, and degradation of all classes of RNAs. RNAs in cells are associated with RNA-binding proteins (RBPs or RNA-binders) to form ribonucleoprotein (RNP) complexes. RBPs influence the structure and interactions of the RNAs and play critical roles in their biogenesis, stability, function, transport and cellular localization [27–30]. RBPs are often subjected to post-translational modifications, enabling them to receive input signals which regulate RNA-binding affinity, signal transduction properties or intracellular distribution of the RBPs [31, 32]. Kinases regulate functions and dynamics of RBPs by influencing protein–protein or RNA–protein interactions in RBP macromolecular complexes [33, 34], or by shuttling between cell compartments [35].

In this work, we provide evidence that the RBP TAF15 is a novel substrate of PKA. PKA phosphorylates one of the TAF15 RNA-binding domains, thereby affecting the binding pattern of RNA species. To delineate the engagement of novel PKA substrate proteins in aberrant cell signaling, our objective was to determine the composition of macromolecular PKA complexes. We identified various RNA-binding and processing proteins as components of macromolecular PKA complexes isolated from colon cancer cell lines which display genetic aberrations leading to cell transformation and uncontrolled cell growth. Our findings suggest that the modulation of RNA–protein interactions, exemplified by TAF15, could represent a novel mechanism by which kinase activities controlled by second messengers can alter post-transcriptional gene regulation.

## Materials and methods

### Cell culture, reagents and antibodies

HEK293T, HEK293 β2AR [36] cells and U87-MG cells (ATCC^*®*^ HTB-14) were grown in Dulbecco’s Modified Eagle Medium (DMEM) supplemented with 10% fetal bovine serum (FBS). The colon cancer cell lines SW480 (ATCC^®^ CCL-228) and SW620 (ATCC^®^ CCL-227) were grown in RPMI-1640 media supplemented with 10% FBS. Cells were grown in water-saturated, 5% CO_2_ atmosphere. Transient transfections were performed with Transfectin reagent (Bio-Rad, #1703352). Forskolin was purchased from MCE Med Chem Express (#HY-15371), Isoproterenol was purchased from Sigma (#I6504), KT5720 was purchase from Enzo Life Sciences (BML-EI199-0100), and employed with indicated concentrations and time frames. Primary antibodies used were the mouse anti-GFP antibody (Roche, #11814460001, Germany), the rabbit polyclonal Phospho-PKA Substrate (RRXS*/T*) antibody (Cell Signaling, #9624), the rabbit polyclonal TAF15 antibody (Cell Signaling, #13150), the mouse monoclonal (mAb) Anti- PKA RIα (D54D9) antibody (BD Biosciences, #610166), and the mouse monoclonal Anti-PKAc antibody (BD Biosciences, #610981). Lamin A/C (Cell Signaling, #4777) Mouse mAb, GAPDH (Cell Signaling, #2118) Rabbit mAb, Phospho-VASP (Ser157) (Cell Signaling, #84519) Rabbit mAb.

### Expression constructs

For TAF15 transient overexpression in mammalian cells, the TAF15 cDNA sequence (NG_023279.1) was inserted as BsrGI/NotI fragment into the plasmid pcDNA3.1 ( +) (Invitrogen) containing the YFP tag cloned as BamHI/BsrGI creating a N-terminal transcriptional fusion and with a 12-aminoacid linker in between. Point mutations at the TAF15 PKA phosphorylation site (S375A, S375E) and partial deletions (ΔRRM, ΔZnF, ΔPY) were introduce by site-directed mutagenesis using the Q5^®^ Site-Directed Mutagenesis Kit (NEB, # E0554). For TAF15 bacterial protein expression, the fusion GST-TAF15 wild-type (wt), S375A and S375E coding sequences were PCR-amplified and cloned into the pGEX-5X-1 vector (Sigma) as EcoRI/XhoI fragments. PKAc-mCherry [37] was used for immunoprecipitation experiments and cell live imaging. All construct were verified by Sanger sequencing. Primer list is shown in Supplementary Table S1.

### Immunoprecipitation

Following 48 h of transient overexpression of indicated YFP-tagged expression constructs, cells were treated with 20 µM forskolin for 15 min, 100 nM Isoproterenol for 15 min or with 5 µM KT5720 for 1 h and then cells were washed with PBS and lysed (standard lysis buffer: 10 mM sodium phosphate (pH 7.2), 150 mM NaCl, 0.5% Triton X-100 supplemented with standard protease inhibitors and phosphatase inhibitors). Cellular debris was homogenized, with 15 strikes of a syringe. The lysate was clarified via centrifugation (13,000 rpm, 20 min) and IPs were performed using Protein A/G mixtures (Invitrogen) and 2 µg of control, anti–flag, or GFP antibodies for 3 h at 4 °C. Resin-associated proteins were washed four times with standard lysis buffer and eluted with Laemmli buffer for Western blot analysis.

### Recombinant protein purificaiton and in vitro phosphorylation assay

GST-TAF15 recombinant proteins were expressed in the *Escherichia coli* strain BL21-DE3-RIL (Novagen) and expression was induced with 0.8 mM isopropyl-β-D-thiogalactopyranoside (IPTG, Sigma #PHG0010) for 16 h at 16 °C. Cells were collected by centrifugation, resuspended in in PBS-0.5% Triton and lysed at 1300 psi using a French press device. Clarified lysates were subjected to GST purifications using Glutathione-sepharose beads (GE Healthcare) following the supplier’s instructions. His6-PKAc expression was carried out in the *Escherichia coli* strain Rosetta pLysS (Novagen) containing the plasmid pET11d-his6-PKAc [38]. Protein expression was induced with 1 mM IPTG for 3 h at 37 °C. Cells were collected by centrifugation and pellets were resuspended in 50 mM sodium phosphate pH 8.0, 300 mM NaCl and 10 mM Imidazol. Clarified lysates were subjected to Ni–NTA agarose purification (Invitrogen) following manufacturer’s instructions. For the phosphorylation reaction, equal amounts of GST-TAF15 protein beads were incubated with recombinant His6-PKAc in phosphorylation buffer (40 mM Tris at pH 7.5, 0.1 mM EGTA, 10 mM ATP and 10 mM MgCl_2_) for 20 min at 30 °C at 1000 rpm. Beads were washed four times with PBS-0.5% Triton, subjected to SDS-PAGE and immunoblotting using an anti-phospho-PKA substrate antibody.

### cAMP-agarose protein precipitation assay

Endogenous PKA protein complexes from SW620/SW480/HEK293 cells were affinity-purified as described before [37]. In short, PKA complexes were isolated under standard conditions without stimulation for baseline binding. Cells were homogenized using a Potter S (B. Braun Biotech International) with 15 strikes (standard lysis buffer: 10 mM sodium phosphate pH 7.2, 150 mM NaCl, 0.5% Triton X-100 supplemented with standard protease inhibitors [PI] and phosphatase inhibitors [PPI]). Cell lysates were clarified (13,000 rpm, 15 min) and endogenous PKA-associated protein complexes were precipitated with PKA-selective Rp-8-AHA-cAMP agarose resin (Biolog, #A012) for 2 h at 4 °C. As negative control experiment, we added excess of cAMP (1 mM) to the lysates to mask the cAMP binding sites in the PKA regulatory subunits for precipitation. Resin-associated proteins were washed four times with standard lysis buffer and eluted with 1% SDS. Finally, resin-associated proteins were subjected to SDS/PAGE followed by immunoblotting, or mass-spectrometry(MS) analysis.

### Phospho-proteome analysis

To determine the composition of affinity isolated macromolecular PKA complexes and characterize the phospho-proteomic contingent, we performed single discovery experiments from samples, which were split in a 80/20 ratio. 20% of the sample was eluted under denaturing conditions (Laemmli Buffer), separated via SDS PAGE and subjected to in-gel tryptic digestion prior to LC–MS/MS analysis. The remaining 80% of the sample were subjected to on-bead tryptic digestion followed by phospho-peptide enrichment using titanium dioxide, prior to LC–MS/MS measurements as described in [39]. Raw MS data was processed and analysed as described in [40]. MS measurements were perform in a Q Exactive HF mass spectrometer (Thermo Scientific, USA). Raw MS data were processed and analysed using Proteome Discoverer 2.1 (Thermo Scientific) with search engine Sequest. The raw files were searched against the uniprot homo sapiens database. Precursor and fragment mass tolerance was set to 10 ppm and 0.02 Da, respectively, and up to two missed cleavages were allowed. Carbamidomethylation of cysteine was set as static modification, oxidation of methionine and phosphorylation of serine threonine and tryptophane as variable modifications. Peptide identifications were filtered at 1% false discovery rate. Mass spectrometry data were generated in DDA mode avoiding any reliable label-free quantification therefore, our approach is largely qualitative and because we carried out single discovery experiments, no classical statistical evaluation was possible.

### Protein network analysis

To create a PPI network of the identified PKA complexes, we superimposed the identified hits on a global interactome library using the STRING database [41] and visualized with Cytoscape 3.0. The network was broken down in multi-protein clusters using an MCL algorithm [42]. These were analyzed for functional enrichment using STRING and grouped accordingly.

### Localization experiments

HEK293 β2AR or HEK293T cells were seeded in a chambered coverslip µ-slide 8-well high (Ibidi, #80806) and transfected with the YFP-TAF15 contructs for 48 h at 37 °C, 5% CO_2_. Live-cell imaging was performed using a Leica TCS SP5 II inverse laser scanning microscope. The settings for colocalization experiments were as followed: GFP was excited at 488 nm using an argon laser and a PMT detector with a spectral range of 500–550 nm. Images were analyzed with the Leica^®^ Imaging Sofware.

### Subcellular fractionation

Cellular fractionation was performed similar to the protocol established in [43]. Briefly, HEK293T and SW480 cells were split into 10 cm dishes and after reaching 80% confluence the cells were treated with 20 µM forskolin for 15 min. Cells were washed in PBS, resuspended in 600 µl hypotonic buffer (20 mM Tris–HCl (pH 7.4), 10 mM KCl, 2 mM MgCl_2_, 1 mM EGTA, 0.5 mM DTT, 0.5 mM PMSF) containing 0.1% NP-40 and incubated on ice for 5 min. After centrifugation (1000 rcf; 4 °C; 5 min) the supernatant (cytoplasmic fraction) was removed and re-centrifuged (15,000 rcf; 4 °C; 3 min) to remove cell debris. The nuclei were washed twice in isotonic buffer (20 mM Tris–HCl (pH 7.4), 150 mM KCl, 2 mM MgCl_2_, 1 mM EGTA, 0.5 mM DTT, 0.5 mM PMSF) containing 0.2% NP-40 and incubated on ice both times for 7 min. After centrifugation (1000 rcf; 4 °C; 5 min) the nuclei were incubated in 100 µl RIPA buffer (50 mM Tris–HCl (pH 7.4), 120 mM NaCl, 1 mM EDTA, 1% NP-40, 0.25% Na-Deoxycholate) for 20 min and then centrifuged (15,000 rcf; 4 °C; 3 min). The supernatant was treated as the nuclear fraction. Laemmli buffer was added to the cytoplasmic and nuclear fractions to subject the samples to Western blot analysis.

### Individual-nucleotide resolution UV crosslinking and immunoprecipitation (iCLIP)

The protocol was performed according to from [44]. HEK293 β2AR cells were plated and transiently transfected with the different TAF15 constructs. After 48 h cells were either exposed to 100 nM isoproterenol for 15 min or directly subjected to cell harvesting. Cells were washed with cold PBS (137 mM NaCl, 2.7 mM KCl, 10 mM Na_2_HPO_4_, 1.8 mM KH_2_PO_4_) and place on ice to be irradiated with 200 mJ/cm^2^ in a UV Stratalinker 2400 (Stratagene). Afterwards, cells were harvested by scraping, centrifuged at 1000 rcf for 1 min at 4 °C, and cell pellets were finally snap frozen on dry ice and stored at − 80 °C till cell lysis. For each sample, 100 μl of protein G Dynabeads (Life Technologies, #161-023) were washed with Lysis Buffer (50 mM Tris–Hcl, pH 7.4, 100 mM NaCl, 1% Igepal CA-630, 0.1% SDS, 0.5% sodium deoxycholate) without protease (PI) and phosphatase inhibitors (PPI), resuspended in 100 μl Lysis Buffer supplemented with PI and PPI and 7.5 μl of GFP antibody (Roche, #11814460001) were added the pre-washed beads, excepting the negative control with no antibody. Tubes were rotated at 4 °C, and afterwards beads were washed with High-Salt Wash Buffer (50 mM Tris–HCl, pH 7.4, 1 M NaCl, 1 mM EDTA, 1% Igepal CA-630, 0.1% SDS, 0.5% sodium deoxycholate), and finally washed twice with Lysis Buffer until the samples were ready. In paralell, cells pellets were resuspended in in 1 ml Lysis Buffer and transfer to a new 1.5 ml RNase-Free microfuge tube (Invitrogen, #AM12450). To digest the cross-linked RNAs, a dilution of either 1:50 or 1:500 RNase I (Invitrogen, #AM2295) was made in Lysis Buffer with futher addition of 10 μl to the lysate together with 2 μl Turbo DNase (Invitrogen, #AM2239) per sample. Then samples were incubated for exactly 3 min at 37 °C shaking at 1100 rpm in a thermomixer and further incubated for 3 min on ice. Lysates were clarified using a Proteus Mini Clarification Spin Column (Serva Electrophoresis, #Gen-MSF500) and subjected to immuprecipitation. For this purpose, the already prepared beads were incubated with the cell lysates for 3 h at 4 °C with constant rotation. Afterwards, the beads were washed twice with High-Salt Wash Buffer, followed by one final wash with PNK Wash Buffer (20 mM Tris–HCl, pH 7.4, 10 mM MgCl_2_, 0.2% Tween-20), and subjected to RNA 3′ end dephosphorylation. To do so, a PNK mix for each sample was prepared with 15 μl water; 4 μl 5X PNK buffer pH 6.5 (350 mM Tris–HCl, pH 6.5, 50 mM MgCl_2_, 5 mM dithiothreitol); 0.5 μl Polynucleotide kinase (PNK, New England Biolab, #M0201L) and 0.5 μl RNasin (Promega, #N2615) and 20 μl of the PNK mix were incubated with the beads at 37 °C for 20 min in a thermomixer at 1100 rpm. Then, samples were washed once with PNK Wash Buffer, another time with High-Salt Wash Buffer and finally washed again twice with PNK Wash Buffer to proceed with the L3 linker ligation. For the linker ligation, beads were resuspended in 20 μl of the following mix per sample: 8 μl water; 5 μl 4X Ligation Buffer (200 mM Tris–HCl, pH 7.8, 40 mM MgCl_2_, 4 mM dithiothreito); 1 μl T4 RNA ligase (New England Biolab, #M0204L); 0.5 μl RNasin; 1.5 μl L3-App linker[44] [20 μM] (IDT); 4 μl PEG 400 (Sigma Aldrich, #81172) and incubated overnight at 16 °C in a thermomixer at 1100 rpm. Next day, samples were washed once with PNK Wash Buffer and twice with High-Salt Wash Buffer. The 5′ end radioactive RNA labelling was performed by adding to the beads 8 μl of PNK mix (6 μl water; 0.4 μl PNK, 0.8 μl 10X PNK buffer, and 0.8 μl ^32^P-γ-ATP [Perkin Elmer, #NEG502A100UC]) with further incubation for 15 min at 37 °C in a thermomixer at 1100 rpm. Visualization of the TAF15:RNA complexes was performed by electrophoretic separation of the samples using NuPAGE and transfer to nitrocellulose membrane. Blots were expose overnight and analysed by Typhoon FLA laser scanner. Afterwards, RNA-TAF15 complexes were isolated by cutting out the region of interest of the nitrocellulose membrane and further digested with Proteinase K (Roche, #311904325) for 20 min at 37 °C in a thermomixer at 1100 rpm. To extract the RNA, samples were incubated with PK buffer-7 M urea (100 mM Tris–HCl, pH 7.4, 50 mM NaCl, 10 mM EDTA, 7 M urea) for 20 min at 37 °C and 1100 rpm and phenol/chloroform extracted using Phase Lock Gel Heavy tubes (VWR, #713-2536) for 5 min at 30 °C shaking at 1100 rpm and separate the phases by centrifugation for 5 min at 16,000 rcf at room temperature. RNA was precipitated using 0.75 μl GlycoBlue (Invitrogen, #AM9515), 40 μl 3 M sodium acetate pH 5.5 and 1 ml 100% ethanol (Merck KGaA, #64-17-5) overnight at −20 °C. RNA pellet was obtained by centrifugation at 21,000 rcf for 20 min at 4 °C, air-dried and further subjected to reverse transcription (RT). RNA was copied into cDNA using Superscript III (Invitrogen, #18080058). Afterwards, RNA alkalyne hydrolysis and precipitation was performed and cDNA was gel-purified in a 6% TBE-urea gel (Invitrogen, #EC6865BOX) to select cDNA library size and eliminate RT primers, further isolated, phenol-extracted and precipitated. The next step was the cDNA circularization, using the CircLigase II (Epicentre, #CL9025K). Then, circularized cDNA was digested with BamHI (NEB, #B7204) and further precipated using GlycoBlue at −20 °C overnight. Finally, cDNA libraries were amplied using Q5 Polymerase (NEB, #M0491) and P3/P5 Solexa primers with specific cycling conditions to ensure proper amplification and PCR products were visualized in 8% TBE-gels. Amplified cDNA libraries were subjected to Illumina Solexa High-Throughput Sequencing (Microsynth, Switzerland).

### iCLIP data analysis

iCLIP reads coming from each experimental group (*N* = 3 independent biological replicates) were phiX-removed, umi-extracted, demultiplexed into samples (4-base inline barcodes), and trimmed (library adaptor removal) using Picard tools (http://broadinstitute.github.io/picard/). Trimmed reads were mapped into the human genome, PCR duplicates were removed, umi deduplicated and counted of uniquely mapped reads and further subjected to normalization as previously described [45]. Differential gene binding sites were extracted from the iCLIP reads of three replicates in each experimental group and statistically assessed to determine fold-change enrichments and p-values. Final iCLIP library read counts were merged by TAF15 phosphorylation motif (S375 and S375A cDNA libraries) or Isoproterenol treatment and analyzed for differential gene expression calculating fold-change enrichments and p-values. RNA feature distribution was assessed using the tool CollectRnaSeqMetrics fromPicard (http://broadinstitute.github.io/picard/). Genomic distribution of mapped reads was visualized using Integrated Genome Browser software [46]. GO biological process analysis was performed using the AmiGO analysis software [47].

### Statistical analysis

Data was tested for Gaussian distribution using the Kolmogorov–Smirnov normality test. Non-Gaussian-distributed data was analyzed using the non-parametric Mann–Whitney *U* test. For Gaussian-distributed data unpaired Student’s *t* test was used to evaluate statistical significance. Values are plotted as means ± SEM. Significance was set at the 95% confidence level and ranked as * (*p* < 0.05), ** (*p* < 0.01), and *** (*p* < 0.001).

## Results

### TAF15 is a novel PKA substrate

**Fig. 1:**
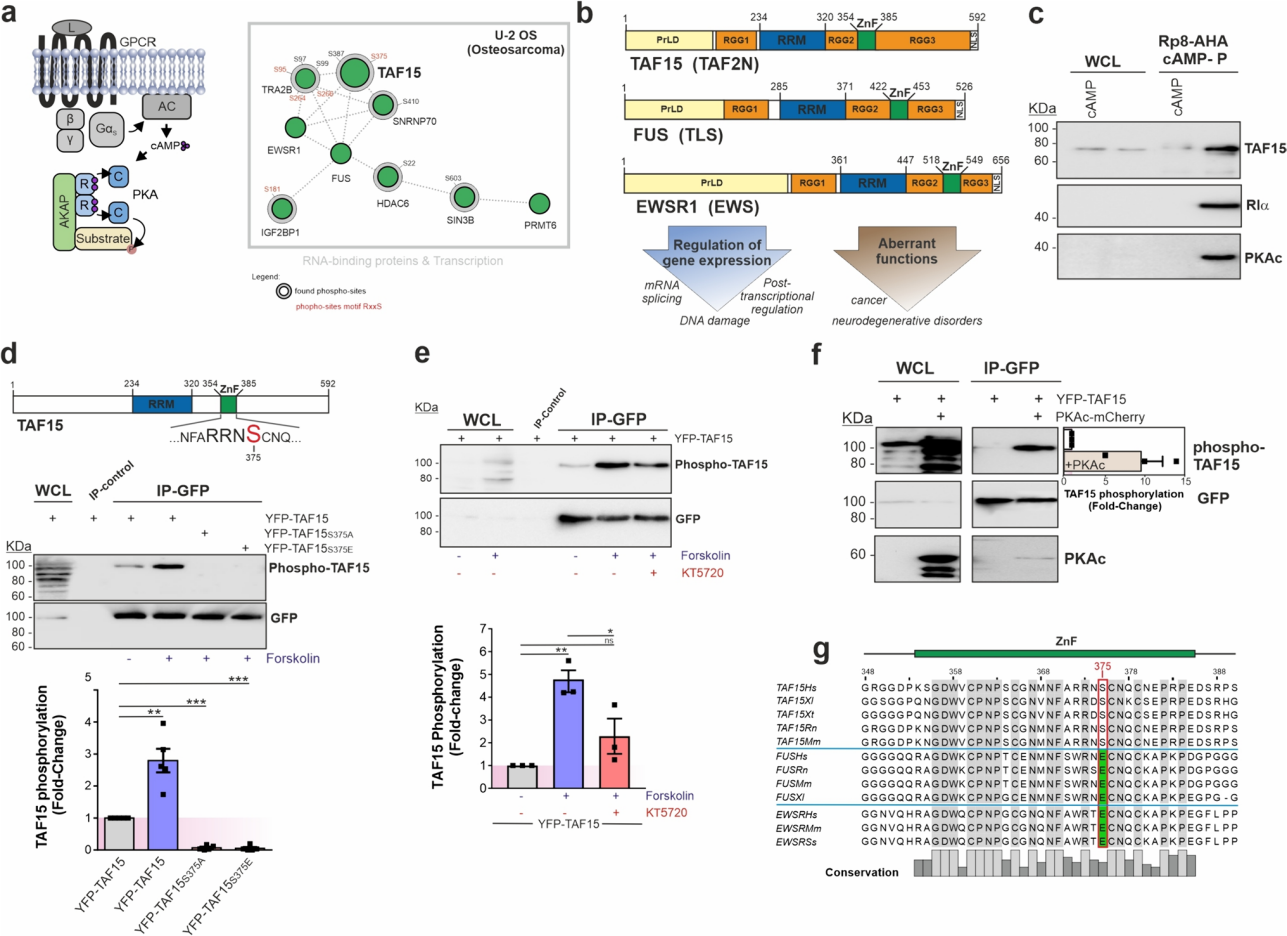
TAF15 is a novel PKA substrate. **a** Left: schematic depiction of the central components of the GPCR-cAMP-PKA signaling axis. Right: PKA interactor sub-network displaying RNA-binding proteins and proteins involved in transcription identified in Bachmann, et al. [37]. The phosphosites are indicated and the PKA motif RxxS-containing sites are highlighted in red. **b** Domain organization of the FET protein family members TAF15, FUS and EWSR1 and major functions in physiological and pathological conditions. PrLD: prion-like low-complexity domain; RGG: arginine-glycine rich domain; RRM: RNA-binding domain; ZnF: Zinc-finger domain; NLS: nuclear localization signal. **c** Western blot analysis of the endogenous PKA macromolecular complexes using Rp8-AHA cAMP precipitation in HEK293 cells. Excess of cAMP (1 mM) was used as negative control. **d** Upper panel: TAF15 domain organization of the two RNA-binding domains (RRM and Zinc-finger, ZnF) and showing the putative PKA phosphorylation site located at the Zinc-finger domain with the phospho-Serine highlighted in red. Middle panel: Western blot analysis of TAF15 phosphorylation using the RRx-S/T phospho-PKA antibody. WCL: whole cell lysate (1% IP input). IP control: IP with Flag antibody. Lower panel: quantification of the phospho-TAF15 signals adjusted to the GFP levels and normalized against the wild-type TAF15 values. Mean ± SEM are shown from five independent experiments (*N* = 5). **e** Upper panel: Western blot analysis following transient expression of TAF15 (wild-type) in HEK293 cells and stimulation with forskolin and pre-treatment with the KT5720 inhibitor (5 µM, 1 h) using the RRx-S/T phospho-PKA antibody. WCL: whole cell lysate (1% IP input). IP control: IP with Flag antibody. Lower panel: quantification of the phospho-TAF15 signals adjusted to GFP levels and normalized against the non-treated control. Mean ± SEM are shown from three independent experiments (*N* = 3). **f** Immunoprecipitation and Western blot analysis of the transient co-expression of TAF15 (YFP-tagged) and PKAc (mCherry-tagged). Quantification of the TAF15 phosphorylation signal is depicted in the corresponding blot, where signals were normalized against the GFP levels and in case of phospho-TAF15 against the mock transfected samples. Values showed are mean ± SEM from three independent experiments (*N* = 3). **g** Protein sequence alignment of the Zinc-finger sequences of the FET protein family members TAF15, FUS and EWSR1. The conserved amino acids are highlighted in grey and the TAF15 phospho-Serine 375 is squared in red showing a replacement for Glutamic acid in FUS and EWS. *Hs* Homo sapiens, *Xl* Xenopus laevis, *Xt* Xenopus tropicalis, *Rn* Ratus norvegicus, *Mm* Mus musculus, *Ss* Sus scrofa. Statistical significance was assessed using an unpaired *t*-test * *p* ≤ 0.05; ** *p* ≤ 0.01; *** *p* ≤ 0.001

In a previous study by our group, we determined a PKA-centered protein–protein interaction network following affinity isolation of macromolecular PKA complexes from the osteosarcoma cell line U-2 OS. In this study we have identified amongst a novel binary interaction partner and AKAP (GPR161) a collection of feasible PKA interactors and substrates which have been linked to RNA metabolism or mRNA processing [37]. Notably, we detected numerous RNA-binding proteins from the FET protein family, with one prominent member being the TATA-box binding protein-associated factor 15 (TAF15, TAF2N), a crucial regulator of gene expression (Fig. 1a). In this phospho-proteomic analysis, we identified multiple phosphopeptides for the PKA phosphorylation site (S375) in TAF15 representing a PKA consensus site for phosphorylation [37]. Building upon this initial observation, we hypothesized that TAF15 represents a novel PKA substrate. TAF15 belongs to the FET protein family, which, in vertebrates, consists of TAF15, FUS (TLS) and EWSR1 (EWS). They interact with a plethora of transcripts, affecting multiple steps in mRNA biogenesis [48]. All members share a conserved modular domain organization (Fig. 1b). They contain a low-complexity domain or prion-like domain (PrLD) relevant for intermolecular complex assemblies. Further, FET proteins display an intrinsically disordered region (RGG domains) acting as regulator of FET nucleotide-protein interactions. In addition, two RNA-binding domains are present, the RRM and Zinc-finger (ZnF) respectively, which mediate the interaction with their RNA targets [48, 49]. Abnormalities of FET protein functions are linked to cancer progression and their nuclear-cytoplasmic shuttling is frequently associated with neurogenerative diseases such as amyotrophic lateral sclerosis (ALS) (Fig. 1b) [48–51]. Our findings underline that PKA may participate in exerting regulatory functions related to RBP-mediated mRNA processing and/or maturation.

First, we aimed to corroborate the coexistence of endogenous TAF15 and PKA in the same macromolecular complex. For this purpose, we repeated precipitations of the endogenous PKA holoenzyme using cAMP analogs (Rp8-AHA cAMP precipitation) in HEK293 cells. We confirmed co-isolations of TAF15 with regulatory and catalytic PKA subunits (Fig. 1c).

Next, we set out to confirm whether TAF15 is phosphorylated by PKA at the phosphorylation motif with the putative phospho-Serine at the position 375, located in the middle of the Zinc-finger domain, one of the RNA-binding domains of TAF15 (Fig. 1d). We conducted IP experiments of transiently overexpressed TAF15 variants in HEK293 cells. We isolated, besides YFP-tagged wild-type TAF15, the non-phosphorylated S375A and phosphomimetic S375E mutants and performed Western blot analyses using an antibody directed against phosphorylated PKA substrate motifs. Our data showed that TAF15 phosphorylation significantly increases upon PKA activation via the cAMP elevating agent forskolin within a short-time frame of exposure (20 µM, 15 min). None of the TAF15 mutants (S375A/E substitutions) were recognized by the antibody, confirming that cAMP-PKA triggers phosphorylation of S375 in TAF15 (Fig. 1d). We further confirmed these observations by in vitro phosphorylation experiments using recombinant proteins. Results showed the respective phosphorylation signal exclusively in the wild-type TAF15 protein in the presence of the PKA catalytic subunit (Supplementary Fig. S1a, b). Furthermore, to confirm the physiological specificity of the PKA phosphorylation on TAF15, we chemically blocked endogenous PKA activity with the selective PKA inhibitor KT5720 prior to inducing cAMP production via the cAMP-elevating agent forskolin (Fig. 1e). The results showed that the elevation of cAMP-mediated phosphorylation was significantly reduced upon KT5720 pre-treatment. Additionally, we analyzed TAF15-S375 phosphorylation upon co-expression of the catalytic subunit of PKA (PKAc) (Fig. 1f). Similar to activation of endogenous PKA, over-expression of exogenous PKAc elevated the phosphorylation of TAF15. Here we also observed that a minor fraction of PKAc co-precipitated with TAF15 (Fig. 1f). Co-precipitation persisted in the PKA substrate-site mutants S375A/E (Supplementary Fig. S1c), suggesting that the interaction is not dependent on the phosphorylation state of TAF15. In summary, our data corroborates that PKA phosphorylates TAF15 at the position S375.

Finally, a sequence comparison of the TAF15 orthologues in other vertebrates showed that the S375 phosphorylation site is well conserved. Surprisingly, it was not present in the other members of the FET protein family FUS and EWSR1. Instead this PKA phosphorylation site is replaced with a glutamic acid (Fig. 1g). We hypothesize that the negative charge of the phospho-serine in TAF15 or the glutamic acid in FUS and EWSR1 could play an important role in the function of FET protein family members. In the case of TAF15, the PKA phosphorylation of S375, located in one of the TAF15 RNA-binding domains, could represent a regulatory mechanism which does not extend to other FET protein members.

### PKA activities alter RNA-binding properties of TAF15

To start to elucidate the functional implications of PKA-mediated TAF15 phosphorylation, we investigated whether the phosphorylation status of TAF15 affects its subcellular localization. Confocal microscopy results showed that neither mutation of the PKA phosphorylation site (S375A/E), nor the complete deletion of the Zinc-finger domain affected the subcellular localization of TAF15 (Supplementary Fig. S2). Similarly, a subcellular fractionation approach showed no alteration of TAF15 localization upon cAMP elevation, in two different cell lines, HEK293T and SW480 respectively (Supplementary Fig. S3).

**Fig. 2:**
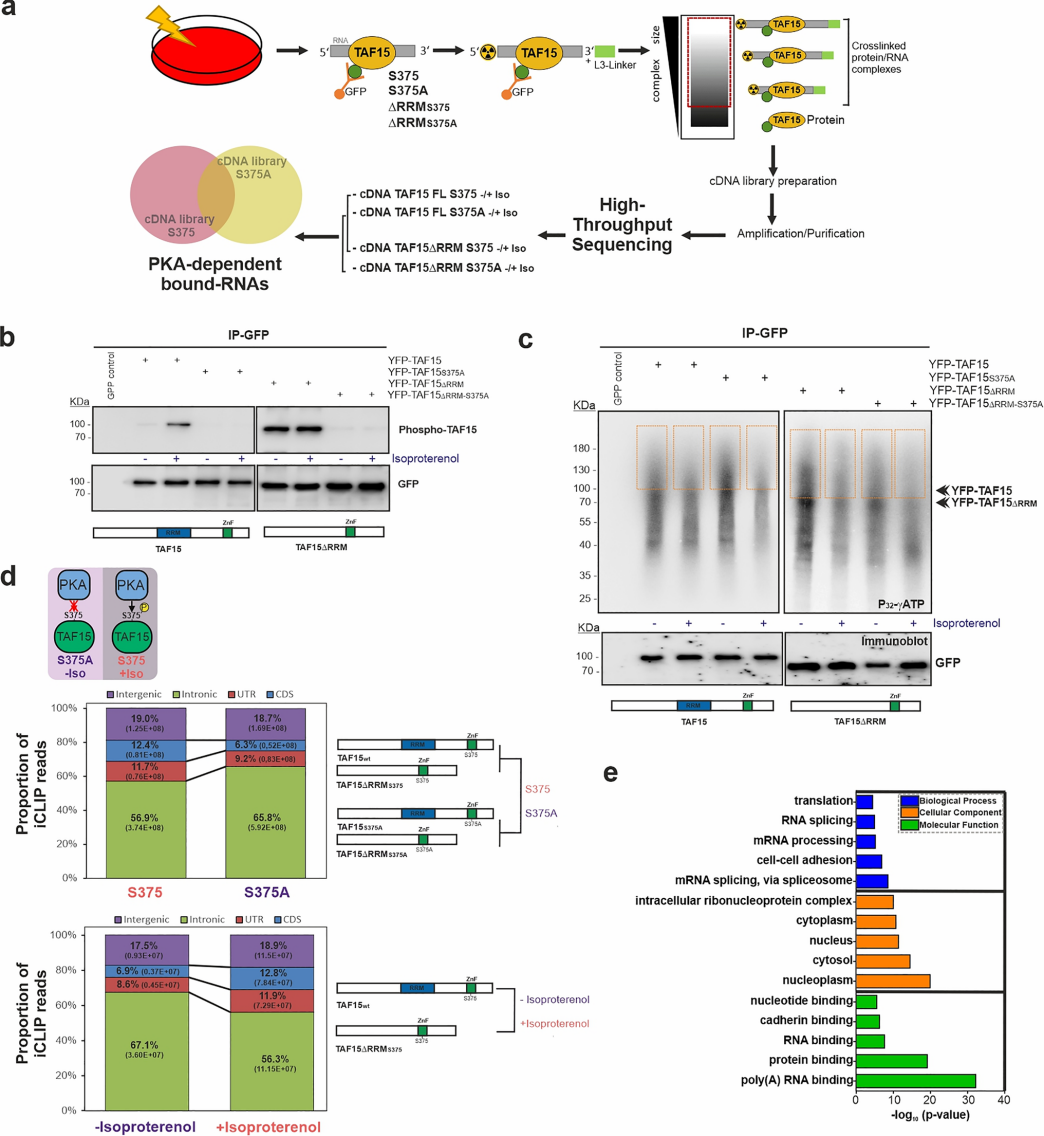
Identification of TAF15 target transcripts regulated by PKA phosphorylation. **a** Schematic depiction of the iCLIP protocol performed in this study [44]. **b** Western blot analysis of the TAF15 constructs subjected to iCLIP. **c** Autoradiograph image of one representative experiment of the TAF15-^32^P-5′ labeled RNA-crosslinked complexes resolved by NuPAGE electrophoresis, blotted on a nitrocellulose membrane, exposed using a Fujifilm radioactive-sensitive film and detected by a phospho-image scanner. Specific complexes above the TAF15 protein molecular weight were excised from the membrane as it is indicated by squares. Western blot analysis of the same samples with α-GFP antibody is shown below the autoradiograph membrane. **d** Genomic distribution of the TAF15-bound targets in TAF15 S375 and S375A (upper panels) and TAF15 S375 − / + isoproterenol (lower panels) iCLIP libraries. Mapped reads were analyzed with Picard to assign the transcript features (CDS, coding sequences; UTR: 5′-3′unstranslated regions; intergenic: other). The comparison of the TAF15 iCLIP libraries is displayed for each case (right panels) and a short depiction of the phosphorylation state of TAF15 on those libraries (above) is shown. **e** Gene Ontology categories of the TAF15 iCLIP read counts significantly impacted by biological process, cellular component and molecular function [47]. Autoradiograph and blots are representative results from *N* = 3 independent experiments

Since the PKA phosphorylation site is located in one of the RNA-binding modules of TAF15, we hypothesized that PKA phosphorylation might alter the affinity of TAF15 for its RNA targets. The TAF15 ZnF is a RanBP2-type zinc-finger which recognizes single-stranded RNA, which generally represents an important aspect of gene regulation [52–54]. For this purpose, and to identify the TAF15-bound RNA species affected by PKA phosphorylation, we performed individual-nucleotide resolution UV cross-linking and immunoprecipitation (iCLIP) experiments [44, 55]. This method allows to analyze protein-RNA interactions on a genome-wide scale for identifying the RNA motif for RNA–protein interactions at nucleotide resolution. The underlying strategy is summarized in Fig. 2a.

To determine changes in the RNA-binding pattern of TAF15 after phosphorylation by PKA, we transiently expressed the YFP-tagged and full-length TAF15 constructs (wild-type and the phospho-mutant TAF15S375A) in HEK293 cells. To exclude possible synergistic effects with the other TAF15 RNA-binding domain (RRM), we included also the TAF15ΔRRM deletion mutants containing the mutation at the PKA phosphorylation site TAF15ΔRRMS375A. Furthermore, to ensure that PKA activation is the driving force of RNA-binding and to get closer physiological context, we exposed the cells to isoproterenol resulting in β-adrenergic-receptor-driven cAMP elevation and activation of PKA. First, to analyze PKA activation we checked the phosphorylation levels of the TAF15 constructs by Western blot analyses (Fig. 2b). We observed the expected increase of TAF15 phosphorylation upon isoproterenol treatment exclusively in the full-length wild-type TAF15, but not in the A-mutant. Unexpectedly, we observed that the ΔRRM mutant exhibited high basal phosphorylation levels when compared to the wild-type full-length, which did not increase significantly upon isoproterenol treatment. Deletion of the RRM domain seems to boost basal TAF15 S375 phosphorylation and renders it insensitive to PKA activation. However, the exact mechanism is unclear.

Subsequently, we initiated the iCLIP experiments. After the UV-crosslinking of TAF15 and their RNA targets in HEK293 cells, we lysed the cells, performed a partial RNase I digestion and isolated TAF15 and its bound RNA species by immunoprecipitation. Crosslinking conditions and RNase digestions have been previously optimized for TAF15 (Supplementary Fig. S4a, b). We labelled the 5′ end of the RNA with radioactive phospho- isotopes, which allowed us to visualize the TAF15-RNA complexes after SDS-PAGE and transfer to nitrocellulose membrane. In contrast to protein-RNA complexes for all TAF15 samples the precipitation using the GFP control did not show as expected any isolations of RNA–protein complexes (Fig. 2c). Major differences of TAF15-RNA complex profiles between TAF15 variants in presence and absence of isoproterenol were not evident. This indicates that neither PKA phosphorylation nor RRM deletion strongly affect the TAF15 RNA-binding capability. Next, we isolated high-molecular weight TAF15-RNA complexes from the membrane and recovered the RNA from the nitrocellulose membrane by digesting the proteins with proteinase K. After RNA isolation we reverse-transcribed the TAF15-bound RNAs into cDNA and we performed a cDNA size selection using gel electrophoresis. We then circularized the purified cDNA and linearized them to finally amplify the linearized cDNA by PCR using 5′-and 3′-Solexa primers. The amplified cDNA libraries were checked by gel electrophoresis (Supplementary Fig. S4c) and were subjected to Illumina deep sequencing.

Analysis of the read counts of the TAF15 cDNA libraries of the S375 wild-type PKA phosphorylation site samples and the mutated S375A samples were filtered and merged to finally get 3334 uniquely mapped reads. Due to the fact that we did not find strong differences between the TAF15 full-length and ΔRMM in the TAF15-RNA complex signals (Fig. 2c), we decided to merge the reads of the PKA phosphorylation sample to finally generate the S375 and S375A iCLIP libraries. After combination, 1307 counts were found exclusively in the TAF15 S375 wild-type libraries, 1306 in the TAF15 S375A and 721 were found in both (Supplementary Fig. S4d), indicating that TAF15 phosphorylation indeed affected the binding specificity and profile of interacting TAF15 RNA populations.

Finally, we analyzed whether the mutation of the PKA phosphorylation site affects RNA coverage distribution. Our data revealed that the S375A mutation sample showed increased binding of TAF15 to RNA targets of intronic regions and decreased the affinities for binding sites in exonic-coding and non-translated regions (UTRs) (Figs. 2d, S4e). Additionally, we compared the binding distribution of the RNA targets in the TAF15 iCLIP libraries containing the S375 wild-type motif and upon cAMP elevation by isoproterenol (Figs. 2d, S4e). Results showed similar distribution of the TAF15 S375 with isoproterenol-treated libraries and S375A with non-stimulated libraries, further validating the approach. Overall, our findings indicate that PKA phosphorylation modulates the binding pattern of TAF15 with its RNA targets by shifting TAF15-RNA interaction sites mostly from intronic to coding regions and, albeit to a lesser degree, UTRs. A GO-enrichment analysis of TAF15-bound transcripts revealed that RNAs with a binding pattern regulated by PKA phosphorylation, are related to RNA maturation, mRNA splicing (poly(A) RNA-binding, mRNA splicing via spliceosome), protein binding and cell–cell-adhesion (Fig. 2e).

### TAF15 RNA targets regulated by PKA

**Fig. 3:**
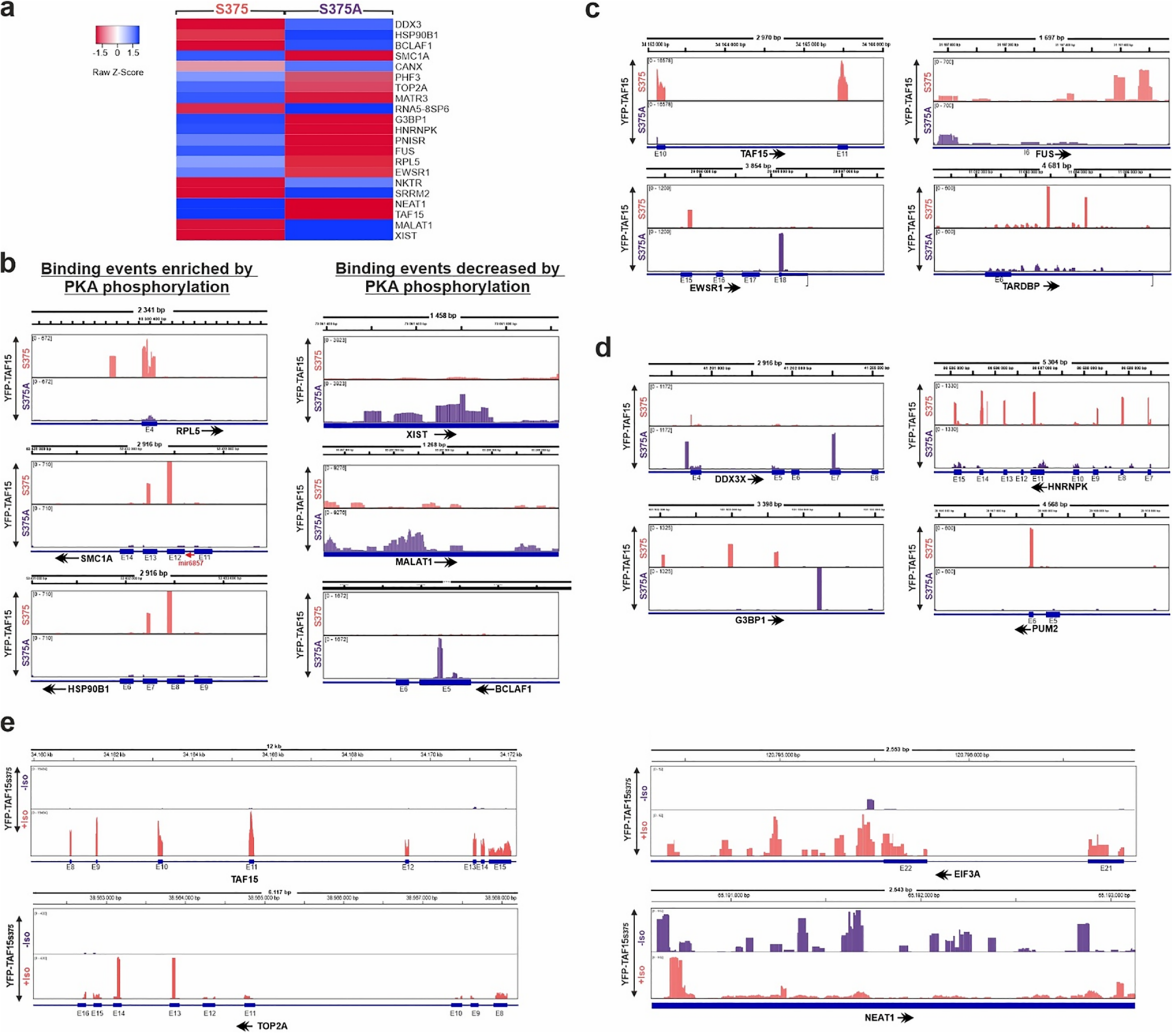
PKA phosphorylation affects RNA-binding patterns of TAF15. **a** Cluster analysis of the most-enriched TAF15 RNA targets of TAF15 S375 (wt) and S375A iCLIP libraries. Z-Scores were calculated and represented using the Heatmapper software [61]. **b** Genome distribution of several examples of TAF15 iCLIP RNA-binding events influenced by PKA phosphorylation. Read count distribution is shown from the TAF15 S375/S375A iCLIP libraries using the Integrated Genome Browser, IGB [46]. Genomic position, annotation and direction of the gene transcription are shown (E; exons, I; introns). Quantification of mapped read counts is shown in brackets in each case. **c** Genome distribution of the read counts from TAF15 S375/S375A iCLIP libraries of FET protein family members using the IGB. **d** Genome distribution of the read counts from TAF15 S375/S375A iCLIP libraries of RNA granule RBPs TAF15 targets influenced by PKA phosphorylation and visualized using the IGB. **e** Genome distribution of the TAF15 iCLIP RNA-binding events in S375 iCLIP libraries under non-cAMP/cAMP elevation by Isoproterenol treatment and visualized by IGB

After analysis of the TAF15-bound RNAs which are modulated by PKA phosphorylation, we analyzed the enrichment of the normalized RNA read counts on S375 cDNA libraries with the S375A (Supplementary Table S2). Interestingly, we found that differentially enriched TAF15 targets, in wild-type vs. S375A libraries, are transcripts of genes involved in regulation of transcription (i.e. BCLAF1, TOP2A, PNISR), FET family protein members (i.e. FUS, EWSR1, TAF15) and transcripts related with stress granule proteins (i.e. DDX3X, HNRNPK, G3BP1) (Fig. 3a, Supplementary Table S3). These findings support the idea that PKA regulates RNA binding pattern of TAF15, switching to transcripts of genes associated with regulation of gene expression and mRNA maturation/splicing. Furthermore, we identified long non-coding RNAs (i.e. NEAT1, MALAT1, XIST), which are described to specifically localize at active transcription sites [56]. Around 22% of the TAF15-bound RNAs we identified in our iCLIP approach were also previously described as targets of TAF15 [57–60].

Afterwards, we closely looked at the distribution of the average peaks across the binding events (RBS) in the TAF15 S375 and S375A iCLIP libraries, to identify reoccurring patterns (Fig. 3b–d). In some cases, we observed that the binding pattern to exons was abolished in transcripts associated with the TAF15S375A mutation (i.e. RPL5, SMC1A, HSP90B1) (Fig. 3b, left panel), indicating that these RBS were enriched by PKA phosphorylation. However, in other cases the binding sites were depleted in the S375 libraries (i.e. XIST, MALAT1, BCLAF1), indicating that PKA phosphorylation depletes these RBS (Fig. 3b, right panel). These observations suggest that PKA might control the affinity of TAF15 for its RNA targets. Interestingly, we could identify that TAF15-bound transcripts of FET protein family members were differentially affected by PKA phosphorylation. In the iCLIP coverage distribution, we observed different binding patterns of TAF15, FUS, EWSR1 and TARDBP in the cDNA libraries (Fig. 3c), suggesting that PKA can also be a key regulator of the binding affinities of TAF15 with other FET mRNAs. Finally, we found transcripts from key stress granules factors (i.e. DDX3, HNRNPK, G3BP1, PUM2) in the PKA regulated TAF15-bound transcript fraction (Fig. 3d). Here, many binding events were shifted from exons to introns in S375A libraries (i.e. HNRNPK), depleted in exon-binding sites (i.e. G3BP1, PUM2), or increased in some regions as observed in DDX3X. Finally, the analysis of the RBS following cAMP elevation conditions corroborated that PKA phosphorylation enhanced the TAF15 binding to exons (i.e. TAF15 and TOP2A mRNAs) and UTR (i.e. eIF3A), and reduced the affinities to non-coding regions) (i.e. NEAT1) (Fig. 3e).

In summary, analysis of binding coverage of the iCLIP libraries shows that PKA phosphorylation modifies binding-pattern and affinities of TAF15 to the majority of its identified RNA targets.

### Phospho-proteome analysis reveals physical links between PKA complexes and RNA metabolism in colorectal cancer (CRC) cell lines

**Fig. 4:**
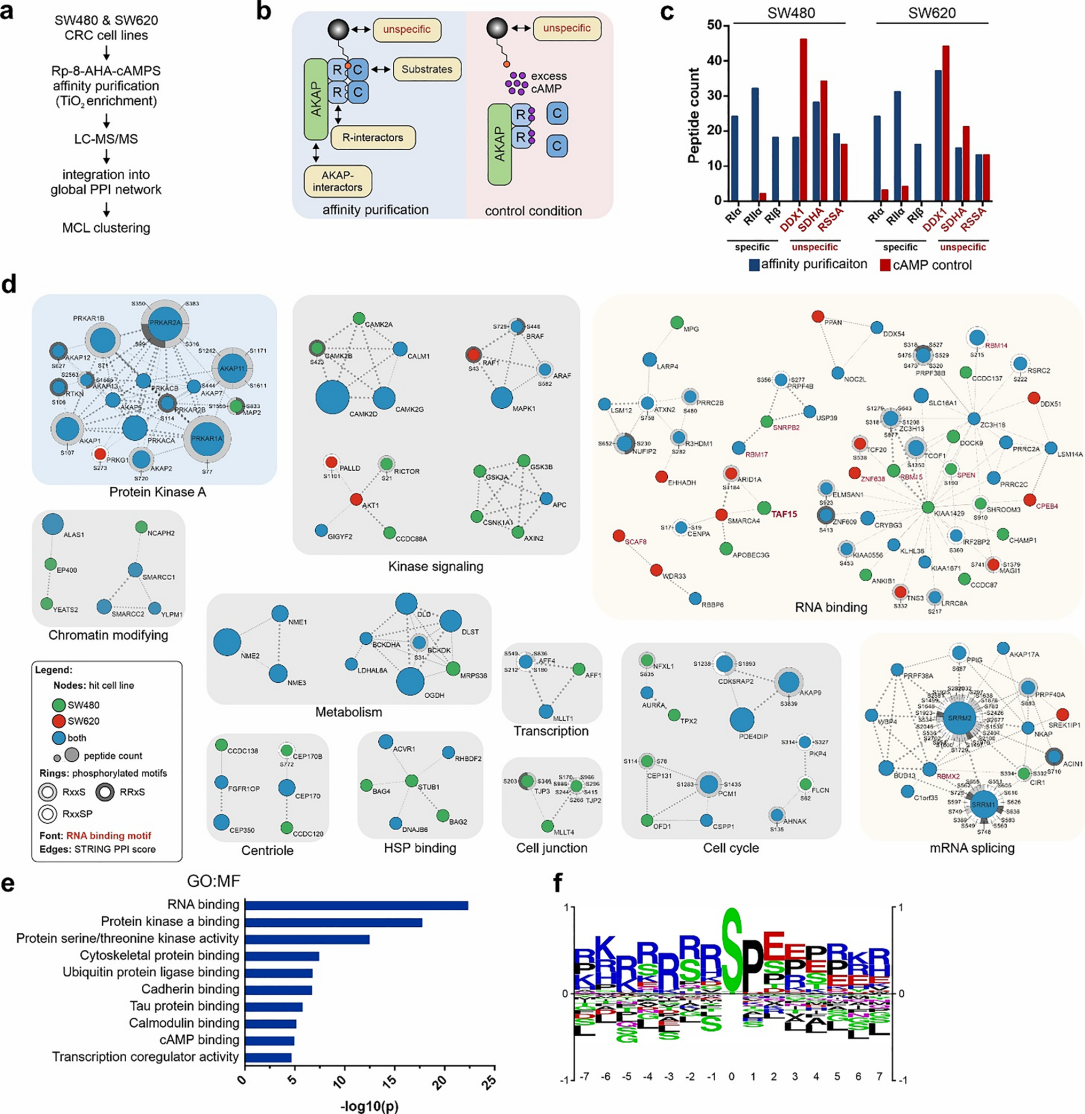
Macromolecular PKA complexes are enriched for RNA-binding proteins in CRC cell lines. **a** Workflow of the applied LC–MS and PPI network analysis. **b** Expected composition of macromolecular PKA complexes isolated via **a** for both, purification- and control condition (excess of cAMP). **c** Comparison of the two conditions for specific binders (highly abundant PKA subunits, black font) and unspecific binders (arbitrarily selected, red font) in both cell lines. **d** Network clusters obtained via workflow shown in **a**. Clusters were tested for functional enrichment and grouped accordingly. Node color indicates cell line specificity, node size indicates the number of identified peptides. Identified phosphorylation of sites matching the PKA consensus motif (RxxS in light grey, RRxS in dark grey and RxxSP in white) are indicated with rings encircling the nodes and labeled with the respective amino acid position. Red labels denote proteins containing RNA-binding domains. **e** Gene ontology (GO) analysis showing selected enriched molecular function terms of affinity isolated proteins. Analysis performed via STRING [41]. **f** Phosphomotif analysis of all detected phosphosites, generated via PhosphoSitePlus [67]

To explore whether other RNA-binding proteins are linked to PKA signaling we determined the macromolecular compositions of PKA complexes from different cell resources. We performed a bottom-up proteomics screening approach, including phospho-proteomic analyses, following label-free affinity-isolation of endogenous PKA complexes from the primary colorectal adenocarcinoma SW480 and its lymph node metastasis-derived derivative the SW620 cell line. We chose CRC cells, as deregulation of the GNAS-cAMP-PKA signaling axis is particularly common in this type of cancer [15, 62, 63]. Similarly as our previous screening, we used the cAMP-analogous Rp-8-AHA-cAMPs agarose affinity resin to isolate macromolecular PKA complexes, followed by liquid chromatography-mass spectrometry (LC–MS/MS) analyses [37], (Fig. 4a, Supplementary File S4). To exclude unspecific binding proteins, we used an excess of cAMP as control condition (Fig. 4b). cAMP competes with the affinity resin for cAMP binding sites and specifically extrudes cAMP binders from the control condition, which is reflected by the peptide count (Fig. 4c). cAMP binding proteins are specifically depleted in the control condition, whereas unspecific binding proteins are not affected. For the detection of phosphorylated peptide entities, we used titanium dioxide enrichment prior to LC–MS/MS measurements. We detected in SW480 cells a total of 607 proteins (166/285 in the cAMP, 156/285 protein binder samples), and in SW620 a total of 454 proteins (226/175 in the cAMP, 53/175 protein binder samples).We matched the identified peptides to the proteome and subtracted unspecific binders to obtain a total of 267 specifically enriched proteins. To generate an integrated PKA-centered protein–protein interaction (PPI) network, we used curated physical interaction information obtained from the STRING database [41]. To improve readability of the resulting network, we segregated the network into clusters via MCL cluster algorithm [42]. Subsequently, clusters were screened for functional enrichment and grouped accordingly, excluding clusters without significant enrichment (Fig. 4d). Again, TAF15 was identified as enriched protein in SW480 cells. We found that clusters enriched for RNA binding and RNA splicing present the largest groups identified, followed by the core PKA complex components. A gene ontology analysis of all 267 proteins shows the highest degree of enrichment for proteins associated with RNA binding, even above protein kinase A binding (Fig. 4e). Lists of all identified peptides, proteins and spectral counts (SPCs) are presented in Supplementary File S4. Several of the obtained hits contained specific RNA-binding domains and are highlighted in Fig. 4d. Besides several members of the arginine/serine-rich splicing factor protein family (SRSF), RNA-binding motif (RBM) proteins family (RBM14, RBM15, RBMX2), mRNA transcription/translation regulators (SCAF8, POLDIP3), and mRNA splicing (SRSF1/SRSF11, TRA2B).

A sequence logo of the identified phosphosites shows that, in line with the well-known target site specificity of PKA [64], we enriched especially arginine-rich sequence stretches (Fig. 4f). Serine was the preferred phosphogroup acceptor with 663 unique phosphorylated sites, followed by threonine with 65 unique sites. Tyrosine phosphorylation was found only marginally, with 6 unique sites. Unexpectedly, we found an enrichment of phosphorylation sites with a proline residue at the + 1 position. While this is a favored target site proline-directed kinase, the typical PKA consensus site contains hydrophobic residues like leucine or phenylalanine at the + 1 position, and proline being strongly disfavored. This might indicate that a large fraction of identified phosphopeptides, present in the isolated macromolecular PKA complexes, could be additionally subjects to phosphorylation by proline-directed kinases [65, 66].

### PKA links to RNA interactions

**Fig. 5:**
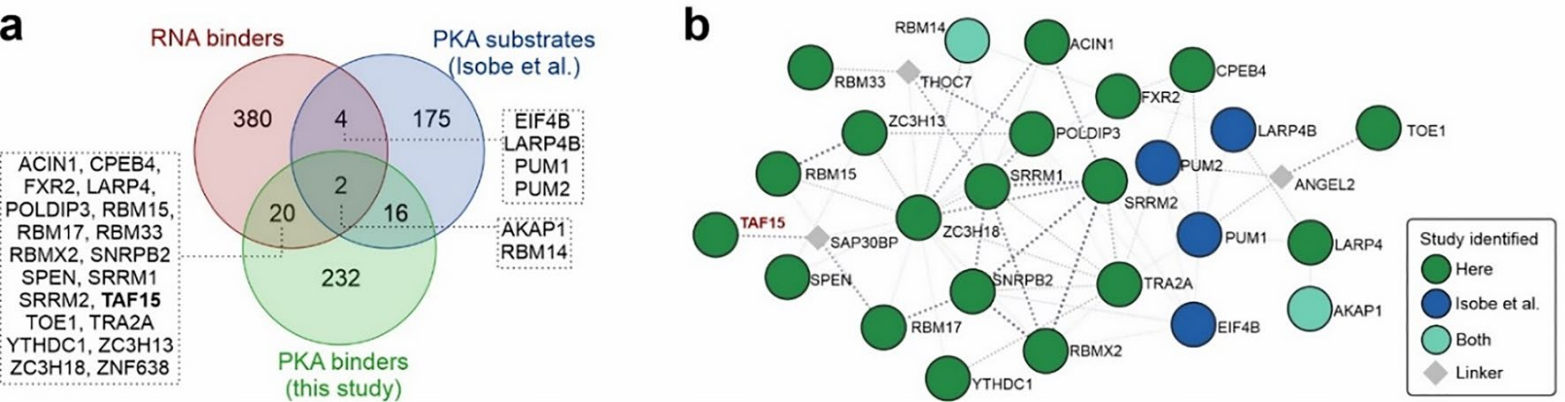
PKA links to RNA-binders involved in mRNA splicing and control of the translation machinery. **a** Venn diagram showing the overlap of PKA targets identified here (green) or in a recent study [68] (blue) with proteins GO-annotated as RNA-binders. **b** Association STRING network [41] of the overlap proteins in **a**

Additionally, we cross-compared the PKA binders we identified in our approach to the PKA protein substrates identified in a recent report [68] and to proteins annotated as RBPs, in the RNA-binding protein DataBase [69]. This analysis showed that six of the proteins which were identified as PKA substrates, and two of which we also identified in our approach, show RNA-binding features (Fig. 5a). Interestingly, most of these candidates are involved in translation regulating functions such as the eukaryotic translation initiation factor 4B (EIF4B), the Pumilio RNA-binding family members 1 and 2 (PUM1, PUM2), and the La ribonucleoprotein 4B (LARP4B). Furthermore, both our studies found the RNA-binding motif protein 14 (RBM14) which is involved in RNA splicing, and finally AKAP1, a classical A-kinase anchor protein. It has been described to recruit the PKA holoenzyme to mitochondria, but is also involved in directing RNAs subcellular compartments [70].

Lastly, analyses of the protein interaction network of these candidates show that most of the identified candidates are interconnected sharing functions related with RNA translation regulation (Fig. 5b), further consolidating the hypothesis that compartmentalized PKA activities are involved in modulating RNA-binding specificities through phosphorylation of RNA-interacting proteins.

## Discussion

Protein kinases are the largest family of evolutionary-conserved proteins acting as key regulators of a plethora of cellular functions such as cell proliferation, metabolism and gene expression. Besides their regulatory functions in cellular signaling, deregulated kinase pathways are frequently associated with several human diseases, particularly cancer, developmental and metabolic disorders [3, 71]. PKA is the prototypical serine/threonine kinase which is widely expressed in all cellular systems and human tissues and represents the classical example of regulation of molecular interactions by phosphorylation of its protein targets.

In this study, we have identified TAF15 as a novel PKA substrate. TAF15 belongs to the FET protein family, which comprises abundant RNA- and DNA-binding proteins that interact with thousands of transcripts and affect multiple steps in mRNA biogenesis. Additionally, they are also involved in the cellular stress response, forming part of the stress granules and the spreading initiation centers [48, 50]. FET proteins are relevant in several different cancers [72] and point mutations in either FUS or TAF15, some of which affect their nuclear–cytoplasmic shuttling, can cause neurodegenerative diseases such as amyotrophic lateral sclerosis (ALS) and frontotemporal lobar dementia (FTD) [73, 74]. The presence of a canonical PKA phosphorylation motif RRxS [75] in TAF15 serves as a unique site of modulation by the kinase. Interestingly, the site is not conserved in the other FET members but is replaced by a negatively charged amino acid (i.e. glutamate), indicating that the local charge in this region might be relevant for the RNA-binding protein function. The possibility to introduce this local charge via phosphorylation of the serine residue might grant TAF15 an additional regulatory layer for fine-tuning of RNA–protein interactions.

The functional implications of reversible protein phosphorylation in fine-tuning mRNA processing are well-established. Phosphorylation of spliceosomal complexes have been described as response to certain cellular stimuli, which cause changes in protein–protein-, RNA–protein interactions, and subcellular localization of ribonucleoproteins [31, 76–78]. We have unveiled that TAF15 phosphorylation by PKA modifies the affinities for several of its RNA targets, suggesting that this modification results in changes of either the conformational landscape or steric interactions between its RNA-binding domains. Surprisingly, deletion of the RRM domain did not have a strong impact in the TAF15:RNA complexes in our iCLIP experiments but changed the phospho-status (Fig. 2c) indicating that its RNA-binding properties are not strictly dependent on the RRM domain. These observations would be consistent with those for FUS, where both RNA-binding domains, RRM and the RGG-ZnF-RGG, bind RNA with similar affinities as the full-length protein [79].

Beyond altering the transcript targeting properties of TAF15, mutation of the PKA site shifts binding towards more intronic regions and cAMP elevation shifts TAF15 RNA-binding to coding regions (Fig. 2d), indicating that PKA phosphorylation might cause TAF15 to preferably bind matured RNAs. This is in line with reports showing that TAF15 mainly binds pre-mRNAs in its basal state [57, 58]. Thus, our data further supports the idea that PKA phosphorylation alters TAF15 RNA binding patterns.

Mutations in the NLS-PY motif have been shown to be present in FET-related diseases such as ALS [80–82]. Cytoplasmic shuttle of FET proteins is a physiological mechanism which is used to regulate stacked mRNAs in stress granules, but these ALS mutations trigger pathologic gains-of-functions of these proteins, resulting in aggregates that are related with progression of the disease [48, 51]. We have further identified that PKA specifically phosphorylates the cytoplasmic TAF15 variant in standard cell lines (e.g. HEK293T cells) and an ALS disease-relevant cell line (e.g. glioblastoma brain cell line) (Supplementary Fig. S5), indicating that this additional regulation layer may be present in ALS pathological conditions. Further analysis of the TAF15 RNAs regulated by PKA in ALS-pathological models would be relevant to identify new molecular mechanisms operating in aberrant TAF15 functions that might provide new insights how kinases control gene expression in neurogenerative diseases. Furthermore, we identified additional, previously reported PKA substrates that are described as RNA-binders (Fig. 5) further supporting the idea that PKA can act as a regulator of RNA-binding specificities. Interestingly, our approach identified another 19 RBP, beyond TAF15, as PKA interactors, several of which contain conserved RRxS PKA target motifs and are feasible PKA candidate substrates.

Initially we identified several connections between PKA and RNA signaling (Fig. 1a, [37]). This observation was further extended in network analyses of endogenous kinase complexes isolated from two CRC cell lines. We have selected two standard CRC cell lines (SW480 and its metastatic counterpart SW620) which are well-studied examples for the activation of the KRAS gene and/or the inactivation of the p53 gene and recently it has been revealed an involvement of mutated trimeric G-proteins in cancer cell progression [62]. Our data shows that PKA might have physical connections with, among others, elements of the transcriptional machinery, spliceosomal proteins and poly(A) mRNA binding, where molecular interactions between RNAs and proteins elements are key mechanisms to control gene expression [83, 84]. Other kinases have been described to coordinate RNA–protein interactions in macromolecular complexes, like CMGC kinases, a well-known example of regulators of RNA–protein complexes by phosphorylating and controlling dynamics of adaptor proteins [85]. In our PKA-centered network, we have identified several proteins well-known to be modulated by PKA, such as the glycogen synthase kinase (GSK), the Ca^2+^/calmodulin-dependent protein kinase II (CAMK2), and elements of the ciliary and centriole complex (i.e. OFD1 and STUB1) [86, 87]. Furthermore, the strong presence of the PKA complex components in our network beyond regulatory and catalytic subunits, like several AKAPs, adds merit to our approach. Our strategy is conceived to systematically identify novel protein–protein interactions and/or compartmentalized substrates emanating from macromolecular PKA complexes. Some kinase-substrate interactions may dissociate upon kinase activation (kiss-and-run) others stay compartmentalized. We used PKA-selective cAMP resin to precipitate the macromolecular PKA holoenzyme to identify compartmentalized PKA substrates that stay physically in the PKA macromolecular complex. However, our strategy appears to also enrich phosphoproteins which might be phosphorylated by proline-directed kinases [66, 88] besides PKA-phosphopeptides, suggesting that both kinase families could be involved. This could also explain the absence of TAF15 as phosphohit in our dataset and might suggest that our approach is better suited to identify specific PKA interactors only at the proteomic, rather than the phospho-proteomic level.

In conclusion, we have identified that the RBP TAF15 is a novel PKA substrate. Our results show that PKA phosphorylation at the TAF15 zinc-finger domain modifies the binding affinities of TAF15 with its RNA targets controlling functions related to mRNA maturation, splicing, protein-binding and other FET mRNAs. Our subsequent search for additional RBPs applicable for this type of regulation revealed several candidate proteins which could be similarly regulated to TAF15. We hypothesize that this finding represents a new mechanism of PKA-mediated regulation of RNA–protein interactions, which could constitute a layer of regulation of gene expression signatures through kinase mediated fine-tuning of RNA–protein binding.

### Supplementary Information

Below is the link to the electronic supplementary material.

Supplementary file1 (PDF 1078 KB)

Supplementary file2 (XLSX 1325 KB)

Supplementary file3 (XLSX 155 KB)

Supplementary file4 (XLSX 1580 KB)

## Data Availability

All data are included in the main text or the Supplementary Materials and can be available upon request. Proteomic data is deposited in PRIDE under the under the project accession PXD048869. iCLIP data is deposited in the European Nucleotide Archive (ENA) under the project number ERP156893.
