# Supplementary material for: Phosphorylation of the compartmentalized PKA substrate TAF15 regulates RNA–protein interactions
Source: Cell Mol Life Sci. 2024 Apr 3;81(1):162. doi: 10.1007/s00018-024-05204-4 (PMC10991009; doi:10.1007/s00018-024-05204-4)
Supplement: Supplementary file 1 — Supplementary file1 (PDF 1078 KB) [file 18_2024_5204_MOESM1_ESM.pdf]

Supplementary Information for

Phosphorylation of the compartmentalized PKA substrate TAF15 regulates RNA-protein interactions

Andreas Feichtner [1,2], Florian Enzler [3], Valentina Kugler[1,2], Katharina Hoppe [4], Sophia Mair [5,6], Leopold Kremser [7], Herbert Lindner [7], Roland G. Huber [8], Ulrich Stelzl [9], Eduard Stefan [1,2] *, and Omar Torres-Quesada [1,10] *

Omar Torres-Quesada; Tel +43 512 9003 70116

Eduard Stefan; Tel +43 512 507 57531

Email: omar.torres-quesada@i-med.ac.at

eduard.stefan@uibk.ac.at

This PDF file includes:

Figures S1 to S6
Table S1

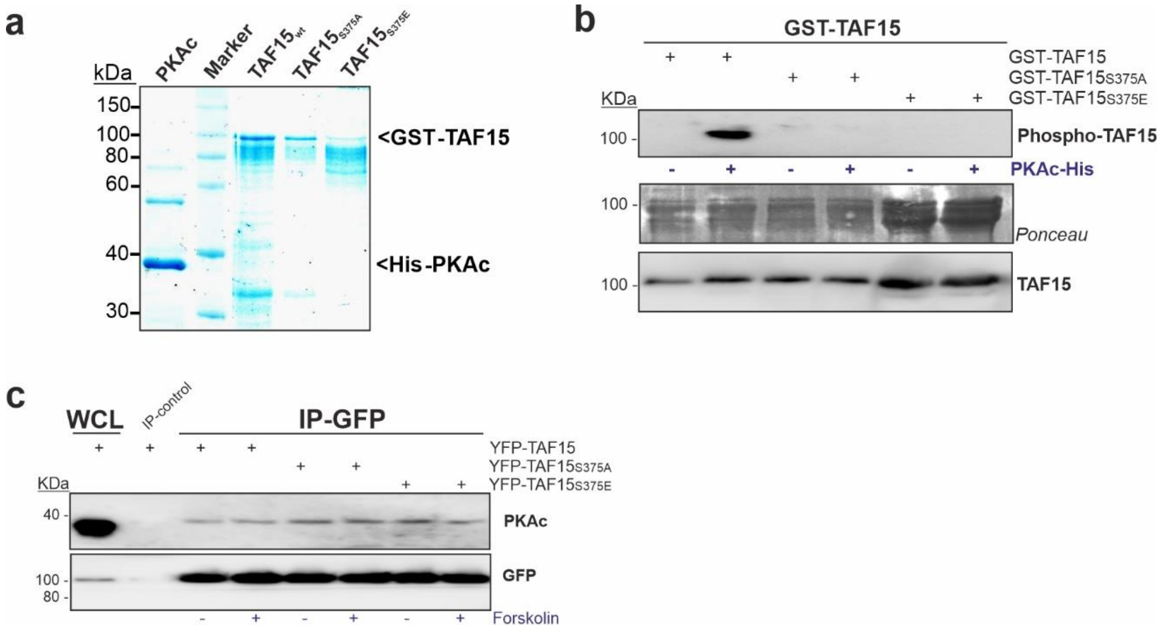

Figure S1. TAF15 and PKA are in the same protein complex but PKA phosphorylation does not influence TAF15-PKA protein-protein interaction. (**a**) Coomasie staining of the **p**urified PKAc-His and YFP-TAF15 recombinant proteins used for in vitro phosphorylation experiments (**b**) In vitro phosphorylation of TAF15 by PKA. In-vitro phosphorylation experiments of recombinant GST-TAF15 purified proteins in the presence of recombinant His-tagged PKAc [1] and using the RRx-S/T phospho-PKA antibody (N=3). (**c**) Western-Blot analysis of the interaction between PKAc-TAF15. HEK cells were co-transfected with PKAc-mCherry and the YFP-TAF15 variants and transiently express them during 48 h. Forskolin-treated samples were performed with 20 µM for 15 min. Cells lysates were subjected to IP with the GFP antibody. WCL represent 0.5% of the IP amounts.

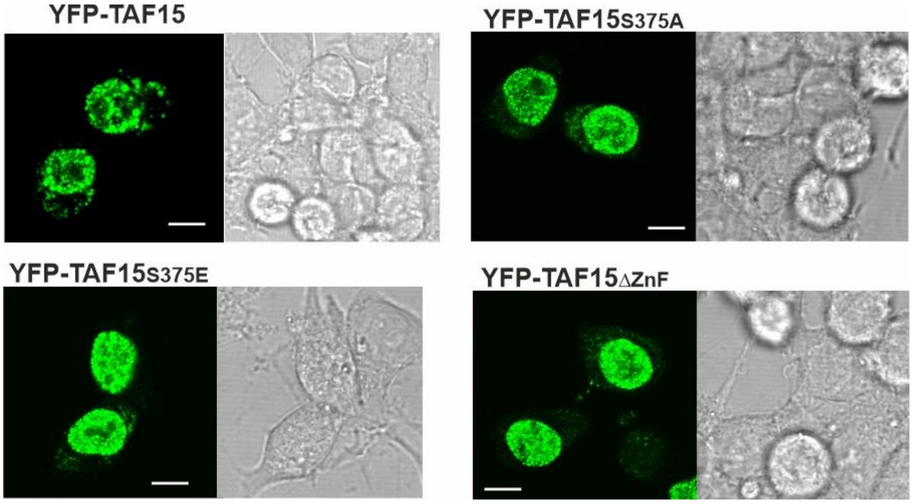

Figure S2. Cellular localization of the YFP-TAF15 variants. HEK293 cells were transfected with the indicated constructs and transiently expressed in a a chambered coverslip µ-slide 8-well high at 37°C, 5% CO_2 for 48h. Live-cell imaging was performed using a Leica TCS SP5 II inverse laser scanning microscope

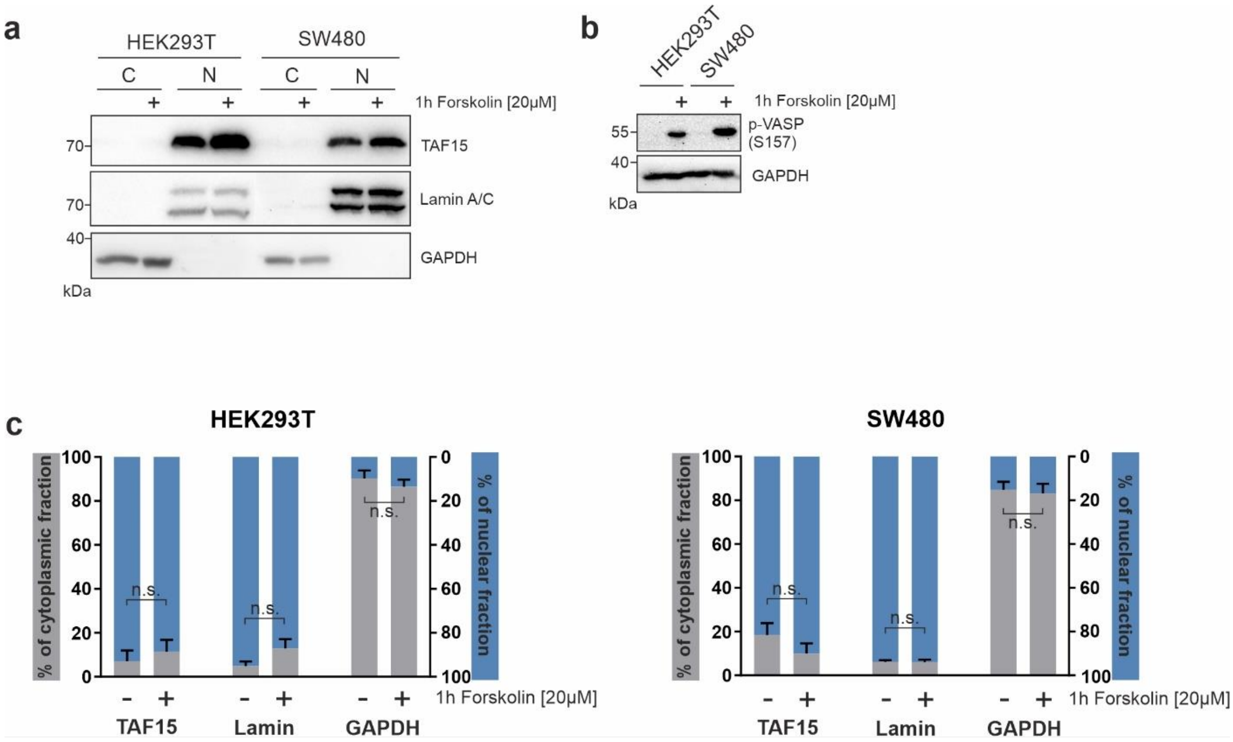

Figure S3. cAMP elevation does not influence TAF15 subcellular localization. Cells, specifically HEK293T and SW480, were cultured in Dulbecco's modified Eagle's medium (DMEM) with 10% fetal bovine serum (FBS) under conditions of 37°C and 5% CO2. Subsequently, upon reaching 80% confluence, the cells were subjected to a 15-minute treatment with 20µM Forskolin. **(a)** Western blot analysis from the cytoplasmic (C) and nuclear (N) fractions. **(b)** Blot corresponding to the confirmation of PKA activation of the Forskolin treatments. **(c)** Quantification of the signals from (a). All representative blots from N=3 independent experiments. Shown are the quantification values (mean ±SEM). Statistical significance was assessed using an unpaired t-student, (n.s.): non-significant.

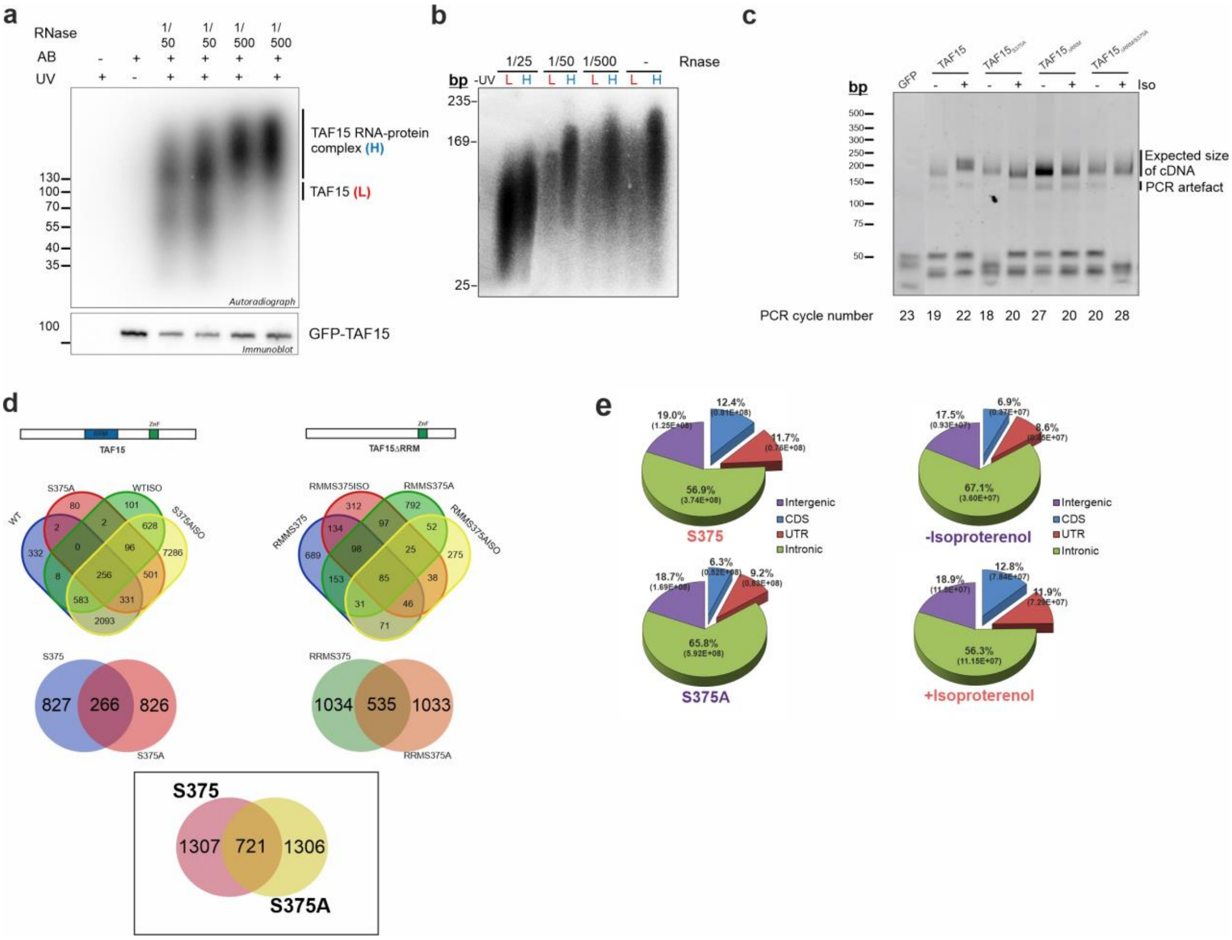

Figure S4. iCLIP optimization experiments and final amplified iCLIP cDNA libraries. (**a**) Autoradiograph of crosslinked TAF15-RNA complexes. The panel shows the autoradiograph of the crosslinked protein-RNA samples as well as the immunoblot against GFP-TAF15. TAF15-bound RNA was isolated from different regions of the nitrocellulose membrane. H; high-molecular weight complexes; L; low-molecular weight complexes. (**b**) RNA electrophoresis of the crosslinked TAF15-RNA complexes under different RNase concentrations. After isolation of the different regions from the nitrocellulose membrane, similarly as in (a), RNA-TAF15 complexes were subjected to Phenol-Chloroform extraction and resolve in a 6% Urea-PAGE gel together with a 23P-labbeled DNA ladder (pUC19/MspI). Gel was dried in a Bio-Rad Gel Dryer Model 543 and expose in a Phospho-screen and detected in a Typhon scanner. (**c**) Final cDNA library amplification of each replicate. PAGE electrophoresis of the PCR amplification of a representative experiment of N = 3 independent replicates is shown. We listed the PCR cycle number. Samples which needed further. Iso: Isoproterenol. (**d**) Analysis of the TAF15 iCLIP read counts. iCLIP libraries of TAF15 full-length and TAF15ΔRRM were merge to finally get the read count number of the S375 and S375A libraries. (**e**) Pie charts of the genomic distribution of the unique mapped reads in TAF15 S375 and S375A (left panels) and isoproterenol treatment (right panels) iCLIP libraries. Mapped reads were analyzed with Picard to assign the transcript features.

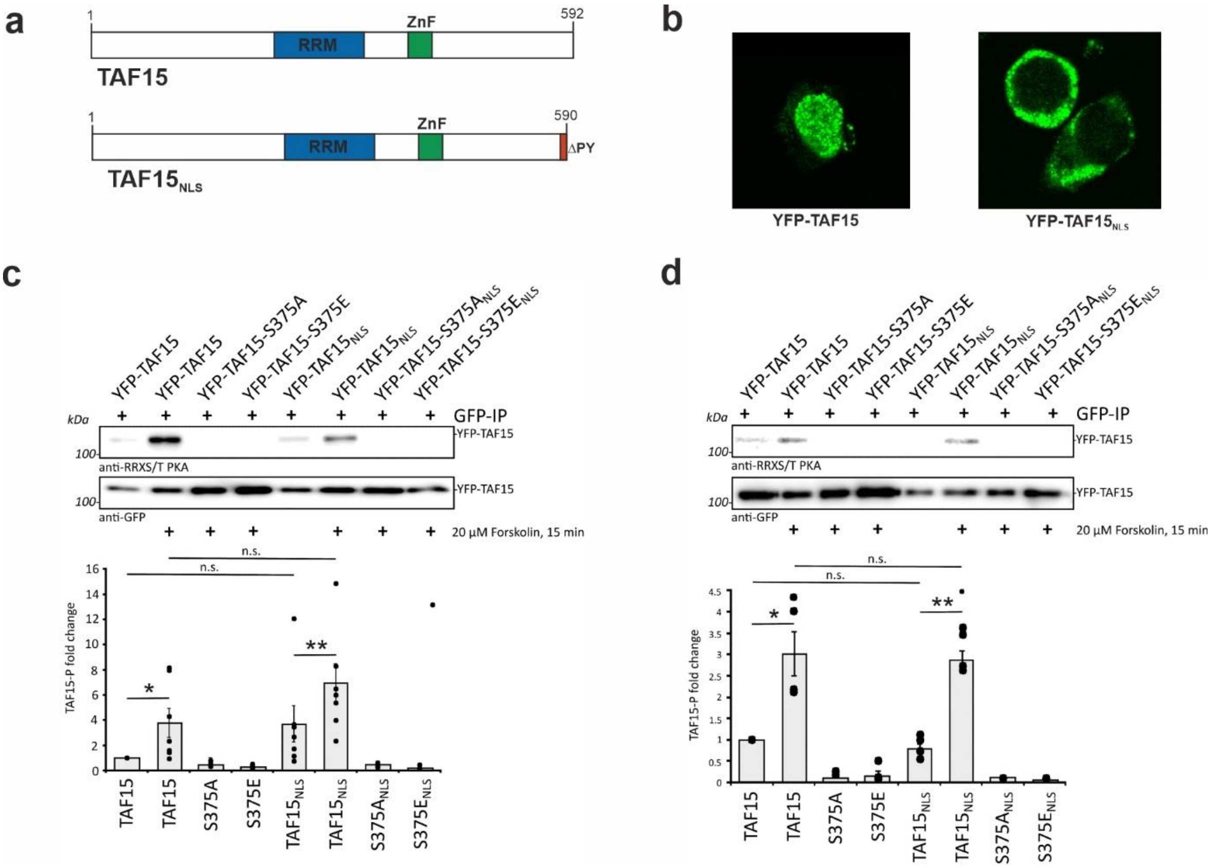

Figure S5. The cytoplasmic TAF15 is phosphorylated by PKA in HEK293T and U87 MG cell lines. (**a**) Schematic depiction of the nuclear TAF15 (above) and cytoplasmic TAF15 (ΔPY mutant, below). (**b**) Confocal microscopy analysis of HEK293 cells transiently expressing the YFP-TAF15 and the YFP-TAF15$_{NLS}$. (**c**) TAF15 phosphorylation experiments of HEK293T cells transiently expressing the indicated constructs. After 48h cells were incubated with forskolin and subjected to lysis and immunoprecipitation using GFP antibody and finally analysed by Western-blot using the RRxS/T phosphor-PKA antibody. Signals were quantified by densitometric analysis and normalized against the GFP levels and the non-treated wild-type sample. (**b**) Same experiments for U87-MG glioblastoma cells. Representative results of N=6 / N =3 independent experiments. Quantifications mean ± SEM. P-values: *=0.05-0.01, **=0.01-0.005.

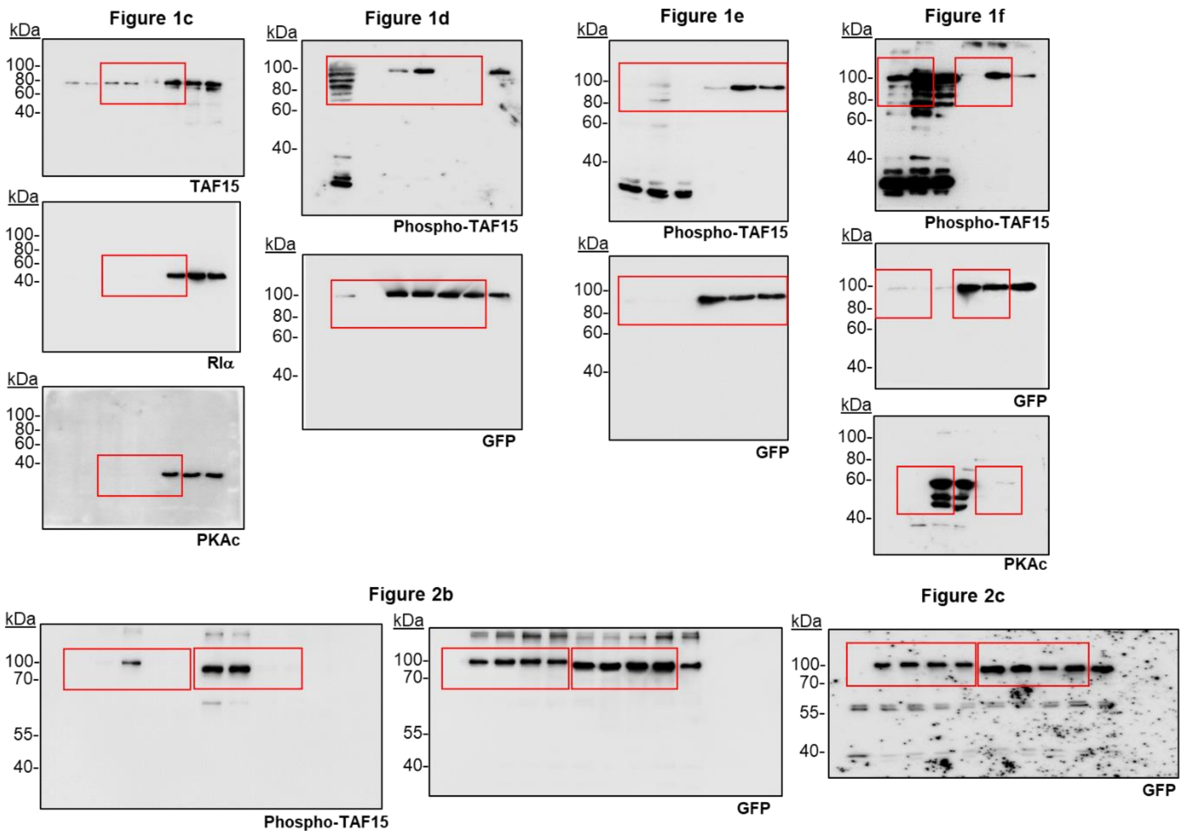

Figure S6. Uncropped gel blots.

Table S1. Oligonucleotides used in this work.

Name	Sequence	Application	Source
YFP-TAF15 Fw	GACGAAGCTTCATGTCGGATTCTGGAAG	YFP fusion	This work
YFP-TAF15 Rv	GATGGATCCGGCCGCGTATGGTCGGTTGCGCTGATC	YFP fusion	This work
TAF15S375A Fw	GAACTTTGCTCGAAGGAATGCCTGCAATCAGTGCAATGAGC	S375A mutant	This work
TAF15S375A Rv	GCTCATTGCACTGATTGCAGGCATTCCTTCGAGCAAAGTTC	S375A mutant	This work
TAF15S375E Fw	GAACTTTGCTCGAAGGAATGAATGCAATCAGTGCAATGAGC	S375E mutant	This work
TAF15S375E Rv	GCTCATTGCACTGATTGCATTCATTCCTTCGAGCAAAGTTC	S375E mutant	This work
GST_TAF15 Fw	AACCGGATCCACATGTCGGATTCTGGAAG	GST purification	This work
GST_TAF15 Rv	GATTGCGGCCGCTCAGTATGGTCG	GST purification	This work
TAF15ΔZnF Fw	GACTCTCGTCCCTCAGGAGGAGATTTC	ZnF deletion mutant	This work
TAF15ΔZnF Rv	GAGGGACGAGAGTCGGGGTCTCCACCTCTCCCTTG	ZnF deletion mutant	This work
TAF15ΔPY Fw	TGAGCGGCCGCTCGAGTCTAGAG	Cytoplasmic mutant	This work
TAF15ΔPY Rv	CGAGCGGCCGCTCATCGGTTGCGCTGATCATTTCTG	Cytoplasmic mutant	This work
L3 - linker:	rApp/AGATCGGAAGAGCGGTTCAG/ddC/*	iCLIP	[2]
RTCLIP_GFP_1	NNAACCNNNAGATCGGAAGAGCGTCGTGGATCCTGAACCGC	iCLIP	[2]
RTCLIP_WT_1	NNACAANNNAGATCGGAAGAGCGTCGTGGATCCTGAACCGC	iCLIP	[2]
RTCLIP_WT_1+ISO	NNATTGNNNAGATCGGAAGAGCGTCGTGGATCCTGAACCGC	iCLIP	[2]
RTCLIP_S375A_1	NNCGCCNNNAGATCGGAAGAGCGTCGTGGATCCTGAACCGC	iCLIP	[2]
RTCLIP_S375A_1+ISO	NNGCCANNNAGATCGGAAGAGCGTCGTGGATCCTGAACCGC	iCLIP	[2]
RTCLIP_RMM_1	NNGACCNNNAGATCGGAAGAGCGTCGTGGATCCTGAACCGC	iCLIP	[2]
RTCLIP_RMM_1+ISO	NNGTGGNNNAGATCGGAAGAGCGTCGTGGATCCTGAACCGC	iCLIP	[2]
RTCLIP_RMM- S375A_1	NNTATTNNNAGATCGGAAGAGCGTCGTGGATCCTGAACCGC	iCLIP	[2]
RTCLIP_RMM- S375A_1+ISO	NNTTAANNNAGATCGGAAGAGCGTCGTGGATCCTGAACCGC	iCLIP	[2]
RTCLIP_GFP_2	NNACGCNNNAGATCGGAAGAGCGTCGTGGATCCTGAACCGC	iCLIP	[2]
RTCLIP_WT_2	NNCGATNNNAGATCGGAAGAGCGTCGTGGATCCTGAACCGC	iCLIP	[2]
RTCLIP_WT_2+ISO	NNCGTANNNAGATCGGAAGAGCGTCGTGGATCCTGAACCGC	iCLIP	[2]
RTCLIP_S375A_2	NNCTCGNNNAGATCGGAAGAGCGTCGTGGATCCTGAACCGC	iCLIP	[2]
RTCLIP_S375A_2+ISO	NNGGCGNNNAGATCGGAAGAGCGTCGTGGATCCTGAACCGC	iCLIP	[2]
RTCLIP_RMM_2	NNGTATNNNAGATCGGAAGAGCGTCGTGGATCCTGAACCGC	iCLIP	[2]
RTCLIP_RMM_2+ISO	NNTGTGNNNAGATCGGAAGAGCGTCGTGGATCCTGAACCGC	iCLIP	[2]
RTCLIP_RMM- S375A_2	NNTTCTNNNAGATCGGAAGAGCGTCGTGGATCCTGAACCGC	iCLIP	[2]
RTCLIP_RMM- S375A_2+ISO	NNAGTTNNNAGATCGGAAGAGCGTCGTGGATCCTGAACCGC	iCLIP	[2]
RTCLIP_GFP_3	NNCTAANNNAGATCGGAAGAGCGTCGTGGATCCTGAACCGC	iCLIP	[2]
RTCLIP_WT_3	NNCATTNNNAGATCGGAAGAGCGTCGTGGATCCTGAACCGC	iCLIP	[2]
RTCLIP_WT_3+ISO	NNGGTTNNNAGATCGGAAGAGCGTCGTGGATCCTGAACCGC	iCLIP	[2]
RTCLIP_S375A_3	NNTCCGNNNAGATCGGAAGAGCGTCGTGGATCCTGAACCGC	iCLIP	[2]
RTCLIP_S375A_3+ISO	NNTGCCNNNAGATCGGAAGAGCGTCGTGGATCCTGAACCGC	iCLIP	[2]
RTCLIP_RMM_3	NAAGGNNNAGATCGGAAGAGCGTCGTGGATCCTGAACCGC	iCLIP	[2]
RTCLIP_RMM_3+ISO	NACGGNNNAGATCGGAAGAGCGTCGTGGATCCTGAACCGC	iCLIP	[2]
RTCLIP_RMM- S375A_3	NAACGNNNAGATCGGAAGAGCGTCGTGGATCCTGAACCGC	iCLIP	[2]
P5 Solexa	AATGATACGGCGACCACCGAGATCTACACTCTTTCCCTACAC GACGCTCTTCCGATCT	iCLIP	[2]
P3 Solexa	CAAGCAGAAGACGGCATACGAGATCGGTCTCGGCATTCCTGC TGAACCGCTCTTCCGATCT	iCLIP	[2]
Cut_oligo	GTTCAGGATCCACGACGCTCTTCAAAA	iCLIP	[2]

1* rApp: pre-adenylated; ddC: dideoxycytidine.
2* RTCLIP primers contain a random barcode sequence (NNxxxxNNN, where N is any base and x the bases of the defined experimental barcode) for the purpose of duplicate removal during sequencing data analysis.

References

1. Porpora M, Sauchella S, Rinaldi L, Delle Donne R, Sepe M, Torres-Quesada O, et al. Counterregulation of cAMP-directed kinase activities controls ciliogenesis. Nature communications. 2018;9(1):1224. doi: 10.1038/s41467-018-03643-9.

2. Huppertz I, Attig J, D'Ambrogio A, Easton LE, Sibley CR, Sugimoto Y, et al. iCLIP: protein-RNA interactions at nucleotide resolution. Methods. 2014;65(3):274-87. doi: 10.1016/j.ymeth.2013.10.011.
